# Criminal reactions to drug-using offenders: A systematic review of the effect of treatment and/or punishment on reduction of drug use and/or criminal recidivism

**DOI:** 10.3389/fpsyt.2023.935755

**Published:** 2023-02-16

**Authors:** Vera Tomaz, Diana Moreira, Olga Souza Cruz

**Affiliations:** ^1^Department of Social and Behavioural Sciences, University of Maia, Maia, Portugal; ^2^Laboratory of Neuropsychophysiology, Faculty of Psychology and Educational Sciences, University of Porto, Porto, Portugal; ^3^Faculty of Philosophy and Social Sciences, Centre for Philosophical and Humanistic Studies, Portuguese Catholic University, Braga, Portugal; ^4^Institute of Psychology and Neuropsychology of Porto – IPNP Health, Porto, Portugal; ^5^Projecto Homem, Centro de Solidariedade de Braga, Braga, Portugal; ^6^Research Centre for Justice and Governance, University of Minho, Braga, Portugal

**Keywords:** recidivism, drugs, treatment, incarceration, offenders

## Abstract

The association between substance use and crime is very common, but complex. Several countries have found strategies to face drug abuse and criminality that may exist associated to it, seeking to reduce overcrowded prisons and to promote the reductions of criminal recidivism and/or substance use. Through the guidelines of PRISMA, a systematic review was conducted with the aim to explore the different criminal reactions to individuals who use substances and are involved in the criminal justice system, namely the role of treatment and/or punishment in the reduction of crime recidivism and/or drug (ab)use. After gathering the following criteria of inclusion (individuals who use substances and are involved in the criminal justice system, between 18 and 65 years old, regardless of gender; consumers of licit/illicit psychoactive substances; without psychopathology not related with use/abuse of drugs; treatment programs; judicial interventions) the database found 155 articles between 1971 and 2022 from which 110 were selected for analysis (57 are from Academic Search Complete, 28 from PsycInfo, 10 from Academic Search Ultimate, seven from Sociology Source Ultimate, four from Business Source Complete, two from Criminal Justice Abstracts, and two from PsycArticles); additional records were included trough manual search. From these studies, 23 articles were included, as they answered the research question, and therefore, constitute the final sample of this revision. The results indicate treatment as an effective response of the criminal justice system in the reduction of criminal recidivism and/or drug use, addressing the criminogenic effect of reclusion/imprisonment. Therefore, interventions that privilege treatment should be chosen, although there are still gaps in terms of evaluation, monitoring and scientific publications regarding the effectiveness of treatment in this population.

## Introduction

In the search for effective responses, within the justice systems, for individuals who use substances and are involved in the criminal justice system, it is important to explore the role of treatment and/or punishment in reducing recidivism and/or consumption of psychoactive substances. So, we conducted a systematic literature review following PRISMA’s guidelines.

The association between psychoactive substances (PAS) and crime, although frequent (1–5), is not linear (3, 5, 6), and there are different interpretations of the phenomenon (7). A single model cannot explain the association between PAS and crime (8), because if PAS leads to crime, then treatment would reduce crime, whereas if crime led to PAS use, then treatment would not be effective and the focus should be on reducing crime (9). However, both the substance-using and the offending populations are heterogeneous, and the paths through which PAS use and crime intersect can be diverse (8).

Furthermore, it is likely that PAS use and crime share risk factors (10), since both criminal behavior and PAS use seem to be related to individual, environmental, psychosocial, developmental, and biological factors (3, 10–12). Crime also exists without PAS, since it appears associated with a set of social forces that potentiate and stimulate it (9). Thus, although crime and PAS are associated, using drugs does not, in itself, entail the commission of crimes (3).

### Criminal reactions to substance-using offenders

Traditional policies to combat PAS, as criminalization, as well as war on drugs, promoted campaigns to eradicate, apprehend and detain all those involved in these contexts (13, 14). As a result of the application of these policies, there was an increase in PAS-related violence and corruption, as well as in the number of inmates, leading to overcrowding in prisons, while the production, trafficking and use of PAS continued, rather than ended. So, alternatives to incarceration began to be studied in these cases. These may be understood as any interventions that aim to: limit the use of incarceration as punishment; reduce the pressure on countries’ criminal justice systems, especially prisons; and decrease the time of deprivation of liberty for substance-related individuals who are also involved in the criminal justice system (13). Thus, incarceration should be used as a last resort and only for high-risk individuals who commit violent crimes (13, 15).

Alternatives to punishment/coercive sanctions for individuals with substance use disorders, namely treatment, education, aftercare, rehabilitation, and social reintegration, can be useful for problems of different orders (16, 17). At the *individual level*—to address addictive problems and reduce the stigma associated with them; at the *social level*—to reduce substance-related problems, especially with regard to acquisitive crimes, as well as to reduce problems associated with public health and other types of damage to society; at the *state level*—to reduce pressure on the criminal justice system and resources used by courts and prisons (17).

Several alternatives to incarceration have been discussed in the specialized literature, frequently appearing grouped and designated in a distinct way. Some alternatives exist only at the theoretical level and the implementation of others occurs in specific contexts. One of the ways to organize different responses of the formal social control system to substance-related crime is to differentiate them into: (i) administrative responses and (ii) responses of the criminal justice system.

Administrative responses are understood as interventions that take place before opening judicial proceedings, in order to prevent entry into the criminal justice system (15, 18), and encourage involvement in treatment (15, 17). These alternatives are associated with three fundamental approaches: decriminalization, depenalization, and pre-detention diversion mechanisms (18).

Decriminalization means that the behavior or action is no longer a criminal offense (13, 19–23). This does not imply that the behavior is legal, since it can be subject to administrative sanctions (21, 22). Therefore, with regard to PAS, behaviors such as possession, acquisition and consumption are no longer considered a criminal offense, although they may constitute administrative offenses and be sanctioned administratively (13, 20, 23). Concerning decriminalization, in some countries, there is a distinction between offenses related to PAS use and offenses associated with PAS possession. So, in some countries, behaviors such as smoking, injecting, inhaling or swallowing PAS are separate crimes from that of being in possession of PAS. As such, in countries like Cyprus, Finland, Spain, Portugal, Armenia and Chile, for example, substance use is a specific criminal offense. Countries such as Spain, Portugal, and Croatia apply non-criminal, and therefore non-custodial, sanctions for PAS use, with preference for the application of fines, suspended sentences, warnings/suspension of proceedings, and/or community work (24). Countries such as Germany, Belgium, Denmark, Slovakia Italy, Poland, and the Czech Republic do not sanction PAS use (20, 21, 25, 26).

Regarding substance possession, Spain, Italy, Luxembourg and Portugal, Estonia, Australia, Jamaica, Mexico, and Peru are examples of countries in which possession of PAS does not constitute a criminal offense (20, 25, 26). However, the definition of criminal offense for substance possession varies according to the legal context of each country. This variation has to do with the possession of: (i) A certain PAS, e.g., in Luxembourg, possession of cannabis resin is not criminally punishable, while possession of other PAS may lead to incarceration; or (ii) any PAS, e.g., in Spain the possession of any PAS in a public place is considered a violation of public safety;—(ii.a) with specific quantities, e.g., in Portugal, the possession of any PAS, above the quantity determined for average individual consumption for 10 days, is defined as a criminal offense; or (ii.b) without specific quantities, e.g., in Italy, punishment does not vary according to the quantity, although exceeding the limits assigned by the Ministry of Health and Justice may be considered possession for sale. In the United States of America (USA), the State of Maryland decriminalized possession of PAS, while the States of California, Connecticut, and Utah consider possession of PAS as a less serious offense (25). Regarding drug trafficking, in the European Union (EU), the sentences applied are preferably prison sentences, most of which are suspended, e.g., Czech Republic and Portugal, and/or community work (Netherlands and United Kingdom) (24).

Depenalization aims to close judicial cases without application of the prison sentence (17), as happens in Germany. The prohibition and criminalization of behavior remain, but incarceration is no longer a strategy to be used, even if other criminal sanctions, e.g., criminal record, are used (18, 21).

The diversion mechanisms available in a pre-detention phase, before criminal charges, include warnings by law enforcement agents, formal bail bonds and procedures involving assessment, education, and/or treatment (15, 17, 27). The warnings/bail bonds are often used in conjunction with referrals to educational sessions, assessment and/or brief interventions or treatment, rather than being charged with an offense. Some of these strategies are applied in countries such as the United Kingdom, Ireland, Malta, and Portugal, and the law enforcement agents, in conjunction with local services, refer individuals with substance use disorders to treatment (17). Thus, a conduct that could be punished with incarceration is diverted to rehabilitation interventions, such as referral to monitoring or treatment systems, or other non-punitive interventions, such as educational interventions (13, 18).

Alternatives to incarceration through the criminal justice system arise in the course of the development of judicial proceedings, varying from country to country, but include the stages of charge/pre-trial, trial/sentence, and post-sentence (15, 16, 18). In the charge/pre-trial phase, police officers and prosecutors have the possibility to decide whether the individual should appear before a court or be referred to alternatives, such as treatment, in cases where they are identified as having a substance-related disorder (13, 15, 16), or referral to other health, and/or social services. Thus, the substance-using individuals involved in the criminal justice system admit their guilt and avoid going to trial (17, 27), with this stage including alternatives such as: suspension of the charge and conditional release in the form of a bail (15, 28). The suspension is applied in cases of first offenses and those of lesser severity, in which the PAS appear to have had an impact on the motivation for criminal conduct. The charge can be suspended upon compliance with a set of conditions, including the termination of treatment, medical and/or psychological, or participation in special treatment programs. Conditional bail can be granted on the condition of participation in treatment. Treatments can be more intensive, such as long-term residential treatment, or less intensive, with release under acknowledgment of the obligations presented (15, 27).

In the trial phase, there may be a deviation from punitive interventions, through legal proceedings such as suspension under judicial supervision (13, 15) and, after conviction, through deviation by substitution mechanisms or reduced sentences, e.g., Probation—alternative measure to incarceration where the judge may choose to suspend the sentence or postpone his/her decision if the offender commits to fulfilling certain conditions (13, 18, 28). Probation involves greater supervision than in suspended sentences and can offer an opportunity to provide psychological, social, and material assistance, as well as to avoid violations of conditions that automatically lead to incarceration, although these approaches depend on the procedures adopted by supervision agencies. In the trial stage, referral to treatment can be used as an alternative, or as a compliment, to punishment (15, 27), depending on whether the sentence is granted or suspended (15, 16). These alternatives are used in less severe cases, when individuals involved in the criminal justice system fail the pre-trial alternatives, when it is unlikely that the offender will commit the offense again, and when treatment is likely to be completed. When the sentence is postponed, the facts are considered proven and sentencing takes place; however, the sentence is not pronounced and is postponed for a certain period of time, during which the offender may be diverted for treatment while under judicial supervision (15, 27). In the event of a sentence suspension, the judge pronounces the sentence, but suspends it for a certain period of time, under certain conditions with which individuals involved in the criminal justice system must comply. There is a declaration of guilt and the measure is added to the criminal record certificate, but there is no deprivation of liberty (15). As such, the punishment is declared, but may be suspended if the offender embarks on a rehabilitative trajectory; this option is available in different countries, such as the Czech Republic, Spain, France, Germany, Lithuania, Luxembourg, the Netherlands, Slovakia, and countries that have Drug Courts (17).

Drug Treatment Courts (DTC) allow the diversion of PAS addicts, from prison to treatment and rehabilitation, by decision of a judge (18, 27). The use of these courts can occur in the post-adjudication/sentence phases, where the offender pleads guilty and the sentence is granted or suspended, so as to allow for a diversion to treatment. After finalizing the court procedures, the sentence is waived or reduced. A diversion to treatment may also take place using DTC even before conviction—admission of guilt is not necessary and the offender is only convicted if he/she fails the completion of the program (13, 15). Contrary to other alternatives in the trial/sentence phase, in the Drug Courts it is the judge who defines the frequency, type, intensity, monitoring, and supervision of the programs; the programs focus not only on disorders related to drug (ab)use, but also on substance-related problems that affect the various domains of the individual’s life; the most intensive treatments are used in the beginning, followed by less intensive treatments in the later stages; and regular follow-up hearings are organized in order to monitor and support prosocial behavior (15). Despite this, DTC have been subject to some critique concerning: the capacity constraints, as the overload of cases (29, 30); the selection bias; the crossover between drug problems and mental health problems; the fact that the need of plea guilt and the criteria for defining the drug disorder and treatment is different among the courts; consequences of program failure; and overriding sentencing laws (30, 31).

In the post-sentence phase, the offender chooses to reduce the sentence of his/her incarceration and be released earlier, under conditional liberty, e.g., parole—early release from incarceration under individual conditions, while undergoing treatment, e.g., the individual must comply with certain conditions, such as treatment for substance addiction and/or social interventions such as education, community work, work/employment programs (13, 15, 27). This means that individuals involved in the criminal justice system may be released after a certain period and/or when a specific part of the sentence has been served, with the offender leaving under individualized post-release conditions and, in the case of substance use, these conditions refer to treatment (15). This type of measure may also include detention; however, it emphasizes the rehabilitation process instead of punishment (27). Examples of this type of measure are supervised treatment programs that aim to address substance-related problems and reintegrate individuals involved in the criminal justice system into the community, for example, community-based treatments (24h supervision) (27).

When an offense occurs, there are mechanisms that can be activated by different justice bodies, in an attempt to divert individuals involved in the criminal justice system from punitive interventions. Specifically, at the police level, authorities can refer detainees to partner entities that work with PAS services, as occurs in Ireland, Malta, and the United Kingdom, or refer them to specific Commissions, as is the case in Portugal. During the prosecution, proceedings may be suspended through PAS awareness courses, e.g., France, motivational interviews, e.g., Norway, and extra-judicial units, e.g., Malta. In court, there may also be suspension of proceedings, suspension of the punitive sentence, or sentencing to rehabilitation interventions and referral to DTC, e.g., Belgium, Ireland, United Kingdom, and Norway (17).

Over time, there has been a concern to gather information and identify intervention principles for this specific population. This concern is also expressed by the Council of the EU, by recognizing the need for Member States to provide and apply, within their legal parameters, alternative interventions to coercive sanctions in the case of substance-using individuals who are also involved in the criminal justice system. These interventions should aim toward preventing crime, reducing recidivism, and increasing the effectiveness and efficiency of the justice system (27, 32–34), taking into consideration the harm reduction in terms of public health and the minimization of social risks (33). It is also important to recognize that substance addiction is a chronic illness (14, 32, 35) that affects behavior. As such, the recovery of addictive behaviors requires effective treatment, followed by continued care (35, 36); thus, services must be adapted to the needs of this specific population. To this end, supervision by the criminal justice system must incorporate the planning of treatment (35).

The EU invites Member States to, whenever possible, offer alternative interventions to punishment or conviction, such as treatment, education, aftercare, rehabilitation, and social reintegration interventions for substance-using individuals who are also involved in the criminal justice system, as well as to identify and develop cost-effective and evidence-based approaches (32, 33). Although incarceration seems to convey the idea of public safety, in reality, it has proved ineffective in reducing recidivism. Specifically, substance-using individuals involved in the criminal justice system are more likely to return to prison, and show no reduction in substance use after incarceration (32, 36). In individuals involved in the criminal justice system with significant PAS-related problems, and whose risk of recidivism is high, treatment programs may be more effective in reducing recidivism than criminal sanctions (27). Thus, these individuals would benefit from alternative interventions that framed these behaviors within a health approach. Such interventions may include, in particular, voluntary treatment for substance-dependent individuals involved in the criminal justice system, community service and referral to health and/or social support services (23). In fact, non-punitive interventions seem to indicate better results than punitive interventions, showing more success in reducing recidivism (18). Specifically, studies suggest that treatment and counseling programs are more effective than incarceration when it comes to reducing addictive behaviors (14).

To conclude, there are currently many cases of substance-using individuals who are also involved in the criminal justice system worldwide which requires the adoption of effective responses to reduce their substance use and criminal recidivism. Therefore, alternatives to incarceration, namely the treatment of PAS use, seem to be effective strategies in reducing not only this practice, but also criminal recidivism, presenting better cost-benefit relationship than incarceration (13, 32, 36). As a matter of fact, these alternative interventions also increase the social inclusion of individuals with substance use disorders (33).

Although there are many approaches related to individuals who use substances and are involved in the criminal justice system, as mentioned above, knowledge about the effectiveness of punitive responses and of alternative responses to punishment, at this level, is still needs more compilation and integration. In addition, through a literature review, no systematic review has yet proposed to understand the criminal reactions to this type of population, as well as the role of treatment and/or punishment as responses of the justice system, in reducing substance use and/or criminal recidivism in substance-using individuals involved in the criminal justice system. Thus, in an attempt to contribute to filling these gaps in theory and seeking to clarify key points in this area, we conducted a systematic review to explore the role of treatment and/or punishment in reducing criminal recidivism and/or the use of PAS in individuals involved in the criminal justice system who use substances. Specifically, we intend to answer the following research question: What is the effect of treatment and/or punishment, as responses of the justice system, in reducing substance use and/or criminal recidivism, in substance-using individuals who are involved in the criminal justice system?

## Materials and methods

This systematic review was conducted in accordance with the PRISMA guidelines (37). The PICO question is included in the title of this manuscript. The main research in databases was conducted on 19 April 2021 but since a systematic review is easily outdated, we conducted a new research on 27 April 2022.

Initially, we defined the research question. Then, we elaborated the best search expression, using synonyms for the variables included in the study and eliminating the terms that had no effect. The search term was introduced in the largest database available in Portugal (i.e., EBSCO, by Faculty of Psychology and Educational Sciences, University of Porto). All abstracts were analyzed by two independent reviewers and disagreements were analyzed by an independent third reviewer. The disagreement (Kappa) was calculated at the stage where there is usually the most discrepancy: the abstract analysis one.

### Study search and selection strategy

The studies were identified using multiple databases from EBSCOhost, namely Academic Search Complete, Academic Search Ultimate, Business Source Complete, Criminal Justice Abstracts, PsycArticles, PsycInfo, and Sociology Source Ultimate. In addition, studies identified through manual search were included, in order to avoid bias and conditioning of the search and information. The search terms were: AB (Abstract) (Incarceration-based drug treatment OR drug treatment OR diversion program OR treatment sentences OR incarceration OR imprisonment OR drug treatment OR drug intervention OR rehabilitation) AND AB (sentence* OR criminal justice programs), AND AB (drug offenders OR addicted offenders OR drug user offenders OR drug misuser offenders OR dependent offenders). The manual search was conducted through a snowball approach, based on the reference lists of the included papers, and through web search using the keywords included in the search terms. The search was not restricted by any linguistic, cultural, or geographical elements.

### Inclusion and exclusion criteria

As suggested by the PRISMA guidelines, studies were selected by two independent reviewers, so as to reduce missed studies or misclassifications (37). In order to choose the studies that would integrate the systematic review, the following inclusion criteria were used: (i) adult individuals involved in the criminal justice system (18–65 years); (ii) individuals who use substances, (iii) treatment programs of any kind, namely, intervention, social reintegration, prevention and/or reduction of recidivism/prevention and/or reduction of drug use, as well as studies on the efficiency of responses/sentences/judicial interventions, and/or (iv) punitive interventions.

The studies were analyzed taking into account the following exclusion criteria: (i) case and/or theoretical studies, (ii) children and adolescents up to 18 years, (iii) exclusive use of pharmaceutical drugs, medically prescribed, and (iv) presence of psychopathology that is not related to substance use/abuse, and/or intellectual disability.

Only articles with empirical and quantitative methodologies, from scientific and academic publications, were included in the study. The disagreement between reviewers was discussed, using an individual excel form and then meet to check out for disagreements, until a consensus was reached. The agreement index was assessed and revealed an almost perfect agreement; *K* = 0.87 *p* < 0.001 (38).

### Identification and screening

**FIGURE 1:**
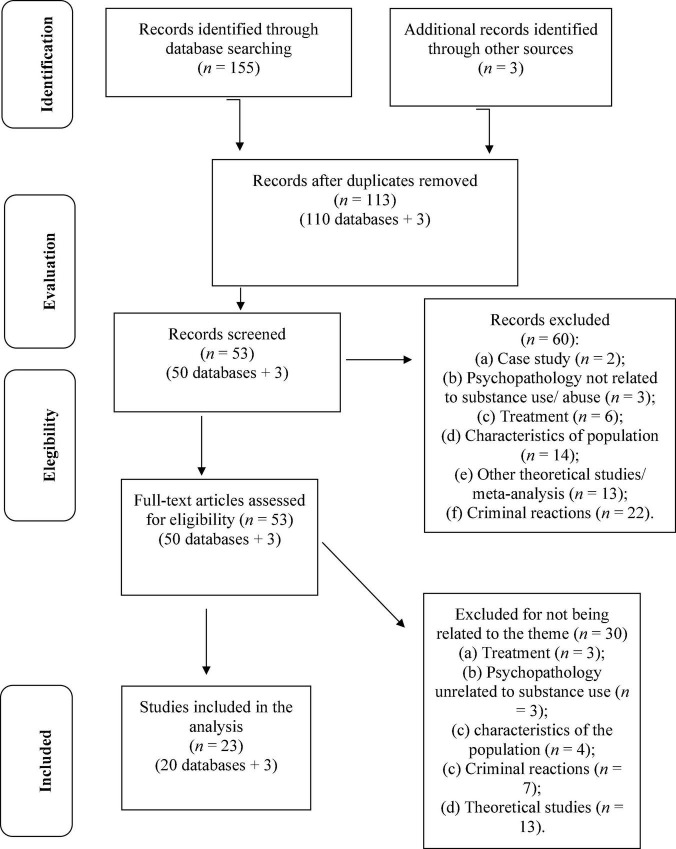
Flowchart of literature search.

A total of 155 studies, published between 1971 and 2022, were identified, through the databases and search methods, and a total of 110 studies were selected for analysis (*n* = 45 duplicates). Of these 110 studies, 57 are from Academic Search Complete, 28 from PsycInfo, 10 from Academic Search Ultimate, seven from Sociology Source Ultimate, four from Business Source Complete, two from Criminal Justice Abstracts, and two from PsycArticles. In the first phase, 60 articles were excluded, since they related to: (a) case studies (*n* = 2), (b) psychopathology not related to substance use/abuse (*n* = 3), (c) characteristics of the population (*n* = 14), (d) other theoretical studies/meta-analysis (*n* = 13), and (e) variables not related to the topic, particularly associated with: (f) treatment (*n* = 6); and (g) criminal reactions (*n* = 22). Of the 50 articles with full reading, three articles were added from manual search. At this stage, 30 articles were eliminated, as they did not answer the research question—specifically due to reasons associated with: (a) treatment (*n* = 3), (b) psychopathology unrelated to substance use/abuse (*n* = 3), (c) characteristics of the population (*n* = 4), (d) criminal reactions (*n* = 7), and (e) theoretical studies (*n* = 13)—and, therefore, do not meet the criteria initially established. Thus, a total of 23 articles were included in this review (cf. Flow diagram of the bibliographic search shown in Figure 1).

## Results

A summary of the characteristics of the studies is presented in Table 1.

**TABLE 1:** Summary of studies’ characteristics.

Study id	Objectives	Sample	Methodology	Conclusion	Context
Anglin et al. (39)	Assess the results of the Treatment Alternatives to Street Crime (TASC)^a^ in five different contexts.	*N* = 2,014 offenders. Offenders who participated in TASC (*n* = 1,114) and who did not participate in TASC (*n* = 900).	Ten critical program elements and Performance standards–Five elected TASC implemented in five states ≠’s. Experimental research design: Canton (*n* = 107 TASC vs. 85) and Portland (*n* = 212 TASC vs. 219); Quasi-experimental research design: Birmingham (*n* = 258 TASC vs. 213), Chicago (*n* = 285 TASC vs. 202), and Orlando (*n* = 252 TASC vs. 219). Substance Use (SU) and Criminal Recidivism (RC) were analyzed through multivariate regression techniques. The assessment of the TASC takes into account three criteria: Treatment services received; SU; CR.	- Provision of services: TASC improves the provision of services offered in 4 of the 5 states. - SU: Efficiency of the TASC in 3 of the 5 states. - Crime: participants in TASC →<probability of being arrested again or technically violating the conditions imposed during the follow-up period. Efficacy of the TASC in 2 of the 5 states. →>supervision >probability of arrest and technical violations of the measure.	Birmingham, Canton, Chicago, Orlando and Portland, USA.
Baird and Frankel (40)	Compare Commonwealth of Pennsylvania’s Department of Corrections (DOC) and Philadelphia (county) Prison (jail) System for offenders who are addicted to alcohol and other substances.	*N* = 37 offenders who use alcohol and other substances (22.6% alcohol addiction). *n* = 15 Stonebridge (Commonwealth of Pennsylvania’s DOC). *n* = 22 New Start (Philadelphia county) Prison (jail) System.	Descriptive analysis of the success of the treatment programs. Programs: both programs offer intensive treatment for alcohol and other substances while promoting the offender’s return to the community. Inclusion criteria: offenders who have a sentence of at least 6 months to complete and who have been identified as having problems related to the use of alcohol or other substances. Substance-related Crimes [SRC] (43.5%), theft (27%), violent crimes (15%).	- Success of the programs: programs demonstrate a 65% completion rate. Stonebridge has >completion rate than New Start (73 vs. 59%). 80% attended therapy 3–5 x a week. More cannabis users (24.3%) completed the program, followed by cocaine users (16.2%). Program completion → efficacy in treating offenders who use alcohol and other substances.	Pennsylvania, Philadelphia, USA.
Banks and Gottfredson (41)	Evaluate the efficacy of 2 of the components [treatment (T) and supervision] of DTC in reducing relapse over 2 years. - Which component (s) contribute(s) most to the reduction of CR.	*N* = 138 (*n* = 49 T: *n* = 36 males; *n* = 40 CRS vs. *n* = 89 w/o treatment). *N* = 138 (*n* = 85 supervision: n = 67 males*; n* = 64 CRS vs. *n* = 53 w/o supervision).	Components: T- multi-phase drug treatment program^b^; supervision—Maryland Division of Parole and probation. CR: Assessed over 2 years using two methods of survival analysis: life-table analysis–to verify the existence of significant differences in the time that elapses until these offenders recur; Cox regression analysis–factors that can contribute to recidivism over time. Three comparison groups: supervision, T, and supervision +T.	- Supervision: 37.6% → do not criminally recidivate (vs. 62.4% who recidivate); - T: 59.2% → do not criminally recidivate (vs. 40.8% who recidivate); - Supervision + T: combination of components → 61.1% do not exhibit recidivism (vs. 38.9% who recidivate); Participants who received the combination of components (T and supervision) >time without recidivating. However, the Cox regression analysis indicated that: - T is the most consistent and significant indicator of the >time without recidivism.	USA.
Belenko et al. (42)	Analyze the impact of DTAP^c^ (Drug Treatment Alternative to Prison) on CR.	*N* = 280 SRC offenders (e.g., selling substances and other allegations of violating the drug law) and non-substance related crimes (NSRC).	Quasi-experimental longitudinal study. - Prospective experimental sample (*n* = 150 DTAP offenders; *n* = 130 prison). Instruments: interviews sociodemographic data, history of substance use and T, criminal history, physical and psychological health, risk behaviors, and social stability; Addiction Severity Index; Michigan Alcohol Screening Test (MAST); Risk Behavior Assessment; Texas Christian University’s Self Rating Form	- Recidivism is < DTAP after 1 year → probability of new arrest is 23% (vs. 45% prison). Robust results over time → 4 years: probability of new arrest = 55% DTAP completed the program (vs. 80% did not complete program). - < possibility of new sentence for all types of crimes: 8% DTAP ↓ new sentence for SRC vs. 18% prison. DTAP ↓ in 56% the probability of new arrest and in 60% the probability of new sentence. DTAP ↓ criminal recidivism (vs. *prison*) especially for those who completed the program.	New York City, USA.
			- Randomly collected retrospective sample (*n* = 64 DTAP completed the program; *n* = 68 DTAP failed it). Instruments: interview applied by the District Attorney’s Office ( = topics of the prospective experimental study sample). Follow-up of 0 to 103 months.		
Brown (43)	Compare repeat offenders/offenders whose charges have been processed by DTC. - Offenders whose DTC has been processed are < likely to recidivate than offenders whose processes are traditionally prosecuted by the justice system between 2004–2006.	*N* = 411 (*n* = 174 DTC, *n* = 107 males) (N/DTC *n* = 274, *n* = 214 males). * In both groups, the offenders met the criteria for admission to DTC.	Databases: - clinical data: Dane County DTC—diagnosis of SU and/or substance addiction disorder; - CCAP^d^: sample characteristics—SRC between 1/01/2004 and 31/01/2006 in DTC (*n* = 137). Cohort study—identify participants with the same characteristics for both groups. For each DTC participant equate 2 offenders other than DTC (*n* = 274). Age, gender, ethnicity, criminal history, and severity index offense, assessed using *t* and chi-square tests. Recidivism: analyzed using Kaplan-Meier and the ′s between curves analyzed using Wilcoxon tests. Survival analyses made it possible to establish a comparison between groups and their characteristics.	- DTC successfully conclude the program →< probability of new crime than N/DTC group (30 vs. 46%, *p* = 0.01). - Average jail time →< DTC than N/DTC (44 days vs. 126 days, *p* < 0.0001). - Time without recidivism →>DTC: *M* days until recidivis*m* → 614 days DTC (vs. 463 days N/DTC). - Time in T: the >the T time →< the probability of recidivism. DTC >time without recidivism in: - offenders with criminal history (felony and prison) (*p* = 0.042); - women (*p* = 0.045); - Offenders over the age of 35 (*p* < 0.001).	Wisconsin, USA.
Dynia and Sung (44)	Assess CR among participants in the T-diversion program applied at the King County District Attorney’s Office on non-violent offenders compared to offenders who did not receive T. - Risk for public safety. - Efficacy of residential T in ↓ criminal k.	*N* = 487 offenders: *n* = 272 decided to participate and were eligible for 1 of 3 residential programs available in DTAP; *n* = 215 did not participate in any program.	CR analyzed in 3 moments in 3 ′s groups: - DTAP concluded the program successfully (*n* = 184); - DTAP did not complete the program (*n* = 88); - N/DTAP (*n* = 170 prison; *n* = 12 jail, *n* = 11 non-custodial measures, *n* = 2 dismissed, *n* = 20 charges dropped). Assessment: 3 years before, throughout the implementation of measures and 3 years later. - Assessment CR: severity of detentions (misdemeanor*^e^* vs. felony*^f^*) and type of offense (SRC vs. NSRC). - Assessment of residential T: Chi squared (differences between pre-detention in the 3 comparison groups) and survival analysis (3-year follow-up).	- Public safety: DTAP (272) → *n* = 12 (4%) detained again (for non-violent offenses). N/DTA (215) → *n* = 28 (13%) detained again (*n* = 23 non-violent crimes, *n* = 3 violent crimes and *n* = 2 misdemeanors); CRS (48% DTAP successful vs. 65% N/DTAP vs. 67% DTAP w/o success). - T: DTAP concluded program → low risk of new arrest during follow-up. 3 years: 23% DTAP successful, detained again (vs. 47% N/DTAP vs. 52% of DTAP did not conclude the program) → DTAP concluded the program →< probability of CR → CR < severity (felony*:* 69% DTAP w/ success vs. 74% N/DTAP vs. 76% did not conclude the program).	Brooklyn, New York, USA.
Evans et al. (45)	To evaluate the effectiveness of SACPA^g^ in offenders on parole and on probation by comparing CR and T success rates.	*N* = 27,208 offenders (*n* = 4,507 parole; *n* = 22,701 probation) treated on an outpatient basis or in residential settings as of 31 December 2008.	Database: CalOMS^h^—characteristics of offenders, history of mental disorders, SU, criminal background, T setting, days in T, and respective success. ACHS^i^ –history of arrests. CR assessed → California Department of Justice (DOJ) through number of arrests after substance T.** Statistical analysis: SAS 9.2/PROC FREQ—comparison of sociodemographic characteristics; Multivariate analysis—sociodemographic comparisons PROC GLM and ≠’s between pre- and post-T in the 2 groups	*-* Parolees: more severe problems when entering T, namely more pronounced SU, polyconsumption and history of previous T program. - Probationers: >outpatient T (86.8 vs. 81.2%); parole >residential T (18 vs. 13.2%). - Probationers →>program completion rate than parole (38 vs. 24%); >T success (37.9 vs. 24.2%). - 12 months: detention rate >parole (53.9 vs. 39.6%). SCAPA seems to be more efficient in probation.	California, USA.
			2 groups under study was assessed using PROC GENMOD. Multiple regression and logistic analyses: short-term results were examined by predictors of the success of T and long-term results were assessed by the number of offenders who were detained again 12 months after T.		
Freiburger and Iannacchione (46)	Understand the effects of incarceration on offenders convicted of SRC and property crimes.	*N* = 413 SRC or property crime offenders and who have been free for at least 2 years.	Collection of research reports between 2000 and 2003 through the Pennsylvania Unified Judicial System^j^ (PSI). Multivariate analysis, independent variables: gender; race; age; profession; education; marital status; w/or w/o children under their care; type of offense (property crimes (*n* = 203) and SRC (*n* = 210); supervision under parole or probation. Control variables: sentenced to incarceration (*n* = 200) (vs. not sentenced to incarceration; *n* = 213).	- CR: 2 variables seem to have an effect on recidivism: age (the older →< probability and severity of CR) and married →< probability of recidivism. - SRC offenders: < probability of more severe crimes. - Incarceration: it is not efficient in SRC and property crimes.	Pennsylvania, USA
Gottfredson and Exum (47)	Assess the BDTC (Baltimore City Division of Parole and Probation) in CR in non-violent, drug-using offenders.	*N* = 235 detainees randomly selected for BDTC (*n* = 139, 74.1% male) or other existing T in the justice system (Control Group [CG] *n* = 96, 74% male).	Sample collected at random from 3 justice units: circuit court and district court supervised by the Division of Parole and Probation and Alternative Sentencing Unit. - CR, assessed: number of arrests, charges, decisions and incarceration time in the 36 months after entering the program. The comparison between groups was analyzed using *t* or chi-square tests, ANOVA was used to verify the interaction between groups. Substance-related issues were assessed in *n* = 49 participants using the Addiction Severity Index.	Addiction Severity Index: 69% → severe problems related to SU (19% severe alcohol problems); 72% → daily use of: crack, cocaine or heroin; 55% → T program history. - CR: BDTC →>probability, statistically significant, sentenced to incarceration or combination of probation and incarceration than the CG. BDTC < probability of new arrest than CG (48.2% BDTC newly arrested vs. 63.5% CG). 1 year later: 57% CG was arrested again vs. 32% BDTC. BDTC reduced in 16% CR. ↓, significantly, probability of new arrests (1.3 BDTC vs. 1.9 GC) and new charges (1.6 BDTC vs. 2.4 CG). BDTC efficient program ↓ CR.	Baltimore, Maryland, USA.
Hearnden (48)	Assess the effects of supervision on reducing SU and crime in offenders on probation.	*N* = 278 offenders addicted to substances and sentenced to probation or another sentence subject to compliance with restrictions.	Sample: gathered by supervision agents from the ILPS^k^: *n* = 278 probationers (men *n* = 226) selected for interview with researcher. 70 of the interviews were collected in: prison, rehabilitation units, T agencies, or semi-public spaces. Impact of supervision, assessed: interviews regarding SU and crimes: 4 weeks before being arrested regarding their substance use and crimes committed and in the last 4 weeks before the interview what is their point of view (that is, 4 weeks after being arrested).	SU changes and crime: - US↓→ 50% maintained heroin use and 37% maintained crack use. - Crime: after entering supervision →↓ in criminal engagement between the 2 moments of assessment—theft in retail stores (from 54 to 17%), selling PAS (from 16 to 5%). ↑ use of legitimate forms of funding: income from labor (18–23%). Offenders whose condition entailed T (*n* = 80 vs. *n* = 180) ↓ Money spent for SU after supervision (513£ vs. 49£).	United Kingdom.
Kearley and Gottfredson (62)	Compare 15-year recidivism between BCDTC or traditional courts.	*N* = 235 offenders random allocated to BCDTC (*n* = 139; 74.1% male) or traditional Courts (*n* = 96; 74% male)	Sample: Maryland Department of Public Safety and Correctional Services; Baltimore Abuse Systems, Inc (BSAS).	Arrests:–32% arrests,–33% charges and—40% property charges than control group; Connections:–27% arrest resulting in one	Maryland, USA.
			Participants randomly assigned. Regression models: group differences; STATA using the MENBERG command: generalization negative binomial model; MIXED command: robustness check.	conviction,–30% total conviction charges,—67% person charges and—42% convicted property charges than control group. Desistance from crime: BCDTC < arrest charges and < conviction charges each year than control group. BCDTC < arrests, charges and convictions in 15-year follow-up period.	
Kim et al. (49)	Understand what factors affect recidivism in SRC offenders and how they react to the conditions of the economic model of crime (incentives and constraints) and the political implications for this type of population.	*N* = 4,398 offenders convicted for SRC (*M* = 0.377 possession; *M*0.381 selling; *M* = 0.092 other SRC^l^).	Proportional model hazard to verify the influence of the 4 determining factors of recidivism: - opportunity-cost (gains obtained as inmates); - disincentives and constraints (generated by the justice system);** - control variables (individual and social characteristics); - duration effects (how the likelihood of recidivism changes with removal from prison). Data were collected through: sentencing guidelines and Florida Department of Corrections Matching Database.	- Offenders on probation →>probability of recidivism (*M* = 1.217 vs. *M* = 0.896 under incarceration). - Incarceration can ↓ PAS use while individual is incarcerated and even after release. But, the >the time away from incarceration, the >the probability of PAS use. SRC offenders seem to respond to incentives and constraints of the judicial system, namely regarding the number of existing police officers (*t* = −2.26).	Florida, USA.
McSweeney et al. (61)	Assess the impact of the Magistrates Early Referral Into Treatment program (MERIT)^m^ on CR.	*N* = 3,319 (MERIT *n* = 1,839) suspects of SRC referred to the MERIT and (N/MERIT *n* = 1,480) offenders who had committed some type of crime (that was not violent and/or sexual) w/ problems related to illegal substances.	Quasi-experimental study: - EG identified through: MIMS^n^- offenders who came from the MERIT in 2008. - CG gathered from: OIMS^o^—individuals addicted to substances identified through application of the LSI-R^p^ and/or convicted between April of 2007 and December of 2008 in a court that did not benefit from MERIT as an option. Descriptive statistics analysis and PSM^q^ + distribution and variance (Kolmogorov-Smirnov) + distribution of categorical variables (chi squared) and ′s between groups (*t-*test). The Mann-Whitney tests was used as a non-parametric alternative when appropriate. Regression models were also used (e.g., Cox).	MERIT → after 1 year: 41.4% MERIT (*n* = 421) convicted again (vs. 36.9% N/MERTI). Conclusion of the program → risk of CR 50% >did not complete the program. SRC: < probability of recidivism than suspects of theft (61%) and other offenses (37%).	New South Wales, Australia.
Miller et al. (50)	How recidivism varies, taking into account the sanction applied in drug offenses. Comparation of incarceration with other types of sanctions.	*N* = 721 (*n* = 686 male; *n* = 263 none criminal history; *n* = 365 sanction type—a fine)	- Register data by the Institute of Criminology and Legal Policy at the University of Helsinki. - Longitudinal panel data—track people from 2009 to 2017 who committed at least one drug offense during 2014. - Logistic regression: analysis the association between sanction type and recidivism. - Matching procedure (1:1 gentic matching algorithm): to minimize the discrepancy and establish robustness. - χ2 test the balance of the variables. Matching using R 3.5.2. - Regression models conducted by Stata 15.1.	General recidivism: >unconditional incarceration (59.4%) compared to conditional incarceration (53.7%) or a fine (54.0%) (differences were not significant). Drug-related recidivism: >unconditional incarceration (29.7%) compared to a fine (27.4%) or conditional incarceration (21.6%). More prior offenses >recidivism (general, 80.6%; drug-related, 41.3%) for both groups; no prior offenses < recidivism (for both groups, significant). For drug-related recidivism, criminal history was the only significant predictor: + prior offenses >recidivism for both groups; no prior offenses < recidivism for both groups, significantly	Finland.
Mitchell et al. (51)	Understand the effects of incarceration on SRC offenders.	*N* = 96,254 (80% male) convicted offenders whose most severe offense is related to substances.	Discontinued regression analysis. Through the database: FDOC^r^ and FDOC’s Offender Based Information System to identify and access all data regarding offenders convicted between 1999 and 2002 in Florida whose SRC was the most severe offense. CR assessed: new conviction and new conviction for SRC during 3-year follow-up.	- According to the CPC (Criminal Punishment Code) only 16% of SRC offenders should receive a prison sentence. - *Prison* ↑ proportion for CR: general in 0.11 (↑ proportion 0.38–0.49); of SRC in 0.8 (from 0.18 to 0.20) in Caucasian offenders. There were no significant ≠’s in CR in SRC offenders in relation to prison → incarceration does not ↓ / ↑ (has a null impact) on CR.	Florida, USA.
Passey et al. (52)	Assess the impact of *Lismore* MERIT^s^ on CR.	*N* = 178 (*n* = 141 males) included during the 18 months of operation of the MERIT (between 1st July 2000 and 31st December 2001). It includes: illegal substance using offenders, w/o violent or sexual criminality.	MIMS—sociodemographic data extraction; NSW Bureau of Crime Statistic and Research—information about convictions and sentencing between 1st January 2000 and 30th September 2001. Survival analysis—measure the 2 ≠’s types of charges: any offense—except infractions against legal proceedings (e.g., bail violation) and theft, robbery, and SRC offenses. For each type of offense, it was calculated the CR between 3 months and 12 months, using the Cox Proportional Hazards analysis. The ≠’s between participants who concluded and did not conclude the program were analyzed according to the Kaplan-Meier.	*N* = 178: >number of charges → crimes against property (e.g., theft, robbery) 30.4 and 24.1% SRC); 53% completed (MERIT w/ success: *n* = 94). *M* days in T: 119 days MERIT w/ success (vs. 57 days MERIT w/o success). - CR (*n* = 175): >MERIT without success. After 3 months: MERIT w/o success →>number of charges for SRC, theft and robbery (30% MERIT w/o success vs. 16% MERIT w/ success); >other offenses (50% MERIT w/o success vs. 25% MERIT w/ success); After 12 months (*n* = 91): MERIT w/ success →< SRC, theft, robbery charges (31% MERIT w/ success vs. 54% MERIT w/o success) and other offenses (53 vs. 69% N/MERIT). - Time elapsed until new offense < MERIT w/ success → MERIT w/o success recidivate more quickly.	Lismore, New Wales, Australia.
Spohn (53)	Compare CR in offenders sentenced to prison and probation.	*N* = 1,077 offenders convicted in the Jackson County Circuit Court in 1993. - Sentenced to prison: *n* = 776 (78.1% male); - Sentenced to probation: *n* = 301 (90.7% male).	Database: Jackson County Criminal Record Information System, with indicators being: - type of offense drug offenders (*n* = 342),	- Prison offenders: >CR than probation: 59.1% were arrested and charged with a new crime during the follow-up period (vs. 33.5% probation). CR index 1.35 (vs. 0.72 probation). >time recidivating (*M* months until new offense: 18.12 prison vs. 33.5 probation). - Interaction type of offense-measure: SRC (drug offenses and drug involved) recidivate more → 4 years: arrested and charged with new offense (65% prison vs. 36% probation) vs. 44% NSRC in offenders sentenced to prison (vs. 37% probation).	Jackson County (Kansas City), Missouri, USA.
Spohn and Holleran (54)	Assess the deterrent effect of incarceration on offenders sentenced to prison or probation. Determine whether incarceration has a ≠’s deterrent effect on SRC offenders than on other types of offenders.	*N* = 1,077 offenders sentenced to prison and probation for drug offenses^t^, drug-involved offenses^u^ and nondrug offenses^v^ in 1993.	Database: Jackson County Criminal Record Information System. Logistic regression analysis. In all multivariate analysis control for: - type of sentence: sentenced to prison (*n* = 776) and probation (*n* = 301); - type of offense: drug offenders (*n* = 342), drug-involved offenders (*n* = 274), and NSRC (*n* = 461);	CR: >in *prison* than in *probation*. - CR time: offenders sentenced to prison recidivate more quickly than on probation. - Interaction sentence-type of offense: prison does not have significant impact on dissuasion in drug offenders. After 4 years: drug offenders prison → probability 5 to 6x >CR than the 3 types of offense on probation. Offenders	Jackson County (Kansas City), Missouri, USA.
			- variables that have an impact on the likelihood of recidivism: gender, race, age, and number of previous convictions.	convicted for drug offenses recidivate more quickly than offenders who commit another offense type. CR—type of offense: >prison (82% drug/prison; 62% drug-inv/prison; 57% nondrug/prison) than on probation (43% drug/probation; 48% drug-inv/probation; 40% nondrug/probation).	
Sung (55)	Assess the effect of DTAP^w^ on CR in substance-using offenders of non-violent crimes.	*N* = 263 substance-using offenders eligible for DTAP between December of 1990 and December of 1992.	Database: DTAP (residential treatment) taking place at Kings County (Brooklyn) District Attorney’s Office. All offenders (*n* = 263) were incarcerated during the admission period. CR, evaluation: incarceration time (3-year follow-up). For those who completed the program, the incarceration time only included the period of admission, for those who failed the program, the incarceration time only included the time of pre-admission, and post-T incarceration. Analysis through: bivariate correlations and logistic regression analysis.	*N* = 263: *n* = 181 successful DTAP; *n* = 81 unsuccessful DTAP and sentenced to incarceration. Follow-up: 30% were rearrested - CR: newly arrested offenders spent more time incarcerated (*M* = 351 days recidivating vs. *M* = 171 non-recidivating) CR →>unsuccessful DTAP (48% unsuccessful DTAP vs. 23% successful DTAP). Probability of being arrested again ↑ with the time the offender spends incarcerated. - T: + time in T →< probability of new arrest (*M* = 404 days recidivating vs. *m* = 566 days not recidivating). T seems efficient in ↓CR.	Brooklyn, New York, USA.
Warner and Kramer (60)	Assess the effects of Restrictive Intermediate Punishments^x^ (RIP/D and A) in the risk of CR in new offense.	*N* = 3,290 offenders sentenced to treatment RIP/D and A between 1998 and 2001 (*n* = 1,552), state incarceration (*n* = 221), county jail (*n* = 892), and probation (*n* = 625).	Regression model (Cox proportional hazards models): compare ≠’s risks of new arrests in participants who received T, sentenced to a state incarceration, county jail and probation. - Dependent variables: new arrest received at the Pennsylvania State Police. - Independent variables: T RPI/D and A, if completed w/ success, the case is closed. Basic characteristics of offenders obtained w/ the database of the PCS (Pennsylvania Commission on Sentencing) and offices of the County Clerk of Courts. Characteristics of the offense: OGC^y^ and PRS (prior record score).	- CR 36 months: new arrests →>offenders sentenced to: county jail (58%), probationers (56%), participants of the RIP/D and A (53%), and state incarcerated (39%). - The successful conclusion of the program considerably ↓ the risk of CR → RIP/D and A w/o success presented a risk 19% >than offenders traditionally sentenced by the system. Risk of new arrest is 61% < RIP/D and A with success. To only be convicted to the RIP/D and A program may not be effective in reducing the risk of recidivism → Successful completion →< risk of recidivism.	Pennsylvania (Allegheny, Berks, Montgomery, Lehigh, Philadelphia, Centre, Cumberland, Lycoming, Schuylkill, Tioga, and Westmoreland), USA.
Weinrath et al. (56)	Compare new charge rates over a 12-month period between DTC group and probation group.	Sample 1: *N* = 199 DTC sample from 2006 to 2014 (*n* = 127 male; *n* = 94 property offense, *n* = 75 drug offense; *n* = 171 criminal history). Sample 2: *N* = 230 (*n* = 63 DTC cases; *n* = 167 probationers)	Sample 1: all cases admitted (graduated or not) in DTC program from 2006 to 2014; SPSS 19: load data and generated descriptive statistics. Sample 2: DTC clients who join the program from 2010 to 2012; Manitoba provincial corrections: access to a 2011 research file of probation admissions (Excel to SPSS); Fisher’s exact test, one-sided and χ2 test: access statistical significance.	Sample 1: 68.3% DTC cases avoid new conviction (85.5% graduated; 59.2% unsuccessful). Graduates 26.3 percentage points >successful avoid new conviction—statistically significant. Complete DTC treatment < reoffence rates. Sample 2: 92.1% DTC cases avoid new serious charge in the 12 months program vs. 79.6% probationers; 81% avoid any new charge vs. 68.9% probation—statistically significant. Charges: DTC < probationers.	Canada.
Yokotani and Tamura (57)	Assess the efficacy of the Personalized Feedback Intervention (PFI)^z^ on illegal substance use T and CR.	*N* = 50 male offenders convicted for SRC who participated in the PFI (*n* = 20 PFI vs. CG *n* = 30).	50 offenders incarcerated between 8th March 2002 and 3rd May 2008; follow-up of 3 years and 6 months. Time without criminal offenses after release—the	*N* = 50: >part sentence for SRC (*n* = 38 US) vs. crimes indirectly related to substances (*n* = 5 robbery, *n* = 4 theft, *n* = 1 rape, *n* = 1 extortion, and *n* = 1 weapons	Japan.
			Kaplan-Meyer method. The adjustment variables that assess the risk of new offense, such as age, incarceration time, number of incarcerations, education were analyzed with the Cox regression model.	law violation). - PFI: ↓ risk SU and SRC →↓ probability of recidivism (25% PFI vs. 40% CG).	
Zanis et al. (58)	Compare the results of the admission of substance-using offenders into Substance Abuse Treatment Facilities (SAFT)^aa^ w/offenders placed on parole w/o T.	*N* = 569 offenders in jail in Northeastern United States.	Eligibility criteria: offenders who have served at least ½ of the sentence; there was still 6 to 12 months of sentence to complete; SU disorder and addictive disorder according to the DSM-III-R; w/o any other disorder unrelated to substances; voluntary participation in the program. Experimental group: *n* = 495 (87, 91% males) SAFT. Level of appropriate care for each offender (assessed by a clinician and psychiatrist through ASAM^ab^ criteria) determined: - IOP^ac^: *n* = 192 (40.4%); - NHR^ad^: *n* = 269 (56.6%). CG: *n* = 74 (13%, 90% male) offenders who were released on parole w/o T subject to standard conditions. Chi squared—parole conditions (without T; with T) and convictions after 24 months of follow-up.	RC 24 months of follow-up: - 22% (*n* = 495) SATF convicted for new crime (vs. 34% N/SAFT, *n* = 74). - *Parole:* w/o T → probability 1.6x >convicted for new crime than w/T. Treatment (*n* = 475): concluded mandatory 6-month treatment (*n* = 178, 37% vs. *n* = 297 w/o concluding T). T completion: 11.8% (*n* = 21) were convicted of new crime (vs. 29%, *n* = 86 w/o concluding T). ↑ number of previous convictions, younger ages and non-completion of the program, are statistically significant variables that seem to predict the possibility of new conviction in a 24-month observation period.	Northeastern city, USA.

^a^TASC—Bridge between criminal justice systems and community-based treatment programs for substance-using offenders. The TASC assesses, identifies and directs offenders to the most appropriate treatment (39).

^b^Includes: Outpatient treatment, methadone maintenance treatment, detoxification, residential treatment, for example (41).

^c^In this study, the DTAP included high-risk offenders, who receive intensive residential treatment (18–24 months), with recidivism being analyzed up to 5 years after treatment or release from prison (42).

^d^CCAP–Consolidated Court Automations Program (43).

^e^Misdemeanor–Minor offenses with a maximum sentence of up to 1 year in prison (https://criminal.findlaw.com/criminal-law-basics/what-distinguishes-a-misdemeanor-from-a-felony.html).

^f^Felony—More serious type of crime with minimum sentence of at least 1 year in prison and maximum of life sentence or death penalty (https://criminal.findlaw.com/criminal-law-basics/what-distinguishes-a-misdemeanor-from-a-felony.html).

^g^Substance Abuse and Crime Prevention Act (SACPA)–adults convicted of non-violent crimes related to substance possession, who are on probation or parole, may be eligible for substance treatment (45).

^h^CalOMS—California Department of Alcohol and Drug Programs (45).

^i^ACHS—Automated Criminal History System (45).

^j^The Pennsylvania Unified Judicial System—website that allows public access to various data related to legal information from different courts (e.g., common pleas, district, and judiciary) (59) (https://ujsportal.pacourts.us/).

^k^ILPS—Inner London Probation Services (48).

^l^Other substance-related crimes are understood in the study as trafficking, distribution, and production (49).

^m^MERIT—Pre-charge diversion program for offenders/adults with proven substance-related problems (current or past). Through treatment, social and health support, the program aims to address the link between substance use and crime, for 3 months (61).

^n^MIMS—MERIT Information Management System (61).

^o^OIMS—Offender Information Management System (61).

^p^LSI-R—Level of Service Inventory-Revised (61).

^q^PSM—Propensity score matching (61).

^r^FDOC—Florida Department of Corrections (51).

^s^Lismore MERIT—Intensive 3-month program, which includes: detoxification, pharmacotherapy, residential rehabilitation, and counseling (individual or group) (52).

^t^Drug offenders—Designated as such when there is considered to be possession or intent of trafficking (54).

^u^Drug-involved offenders—Previous history of substance abuse or previous drug offense conviction (54).

^v^Nondrug offenders—convicted for crimes against property (54).

^w^DTAP—For offenders who completed treatment, the charges were dropped; for those who did not complete treatment, the charges proceeded and they were sentenced to incarceration (55).

^x^RIP—Restrictive Intermediate Punishment: introduction of intermediate punitive measures as an alternative to incarceration. Examples of RIP sanctions include: treatment of alcohol and other substances (Danda), house arrest with an electronic bracelet, or boot camps.

^y^OGC–measures the severity of the offense (60).

^z^(PFI) Personalized Feedback Intervention—Indirect treatment method (vs. personal contact) that combines motivational interviewing techniques with information based on risk factors, as well as relapse prevention skills training (57).

^aa^SAFT—Early release from jail directly to a substance treatment unit allowing the offender to complete the sentence in these units (58).

^ab^ASAM—American Society of Addiction Medicine (58).

^ac^IOP—Intensive Outpatient Program (58).

^ad^NHR—Non-Hospital Residential (58).

### Drug-using offenders

There are characteristics that prove to be more common among substance-using individuals involved in the criminal justice system. Specifically, most are male (44, 49, 52, 56, 58, 60), unemployed (46, 48, 52), and have already undergone some type of treatment (48, 47). Mostly, these individuals involved in the criminal justice system are single (40) and ages between 29 and 31 years (40, 52, 58).

Regarding substance use, these individuals involved in the criminal justice system initiate this practice during adolescence (48), specifically before the age of 20 (40), and justify it with personal issues and the relationship they maintained with other individuals who use substances (48). The most popular PAS are heroin (40, 47, 48, 52), cocaine hydrochloride (40, 47, 56, 58), alcohol (40, 47, 58), crack (40, 47, 48), cannabis, and amphetamines (52).

Recidivism appears to be affected by variables such as gender, age (43, 46, 54, 58), criminal background (43, 46, 50, 54, 58), marital status (46), and type of offense (61). Specifically, men seem to be more likely to recidivate (49, 54, 60), and to engage in more severe forms of recidivism (46). Younger people recidivate more (54) and, therefore, as age increases, the probability of recidivism seems to decrease, regardless of gender (60). Married people are less likely to recidivate than unmarried people (46), and individuals involved in the criminal justice system with previous convictions have higher rates of recidivism (54, 58).

Regarding criminal background, most substance-using individuals also reveal histories of problems with justice since adolescence (48), thus the age of the first arrest appears to be related to the age they first started using substances (40).

### Recidivism

#### Incarceration and probation

The results on incarceration indicate that this variable seems to have little impact on the reduction of criminal recidivism (46, 51, 53), since it does not effectively prevent the development of criminal behavior. There is no evidence that individuals sentenced to incarceration delay their re-entry into crime; and incarcerated individuals have a recidivism rate of 1.35 vs. 0.72 of those in probation (53). The probability of a new arrest increases with the time the offender spends in prison (55).

Data on the deterrent effect of incarceration does not seem to reach consensus. In some studies, incarceration seems to have a deterrent effect on individuals involved in the criminal justice system with better social integration (46, 51), and it is possible to verify that incarceration seems to have a greater deterrent power than probation (*M* = 1.217 probation vs. *M* = 0.896 incarceration) (49). For this reason, these individuals seem to respond to the incentives and constraints of the justice system, namely the number of police officers that exist in the surrounding areas (49). Miller et al. (50) study points that for drug-related recidivism, criminal history was the only significant predictor compared to no prior offenses (one prior offense: *OR* = 2.04, *p* < 0.05; 2–5 prior offense: OR = 3.01, *p* < 0.001; more than 5 pior offenses: OR = 5.33, *p* < 0.001). In Finland, incarcerated individuals do not manifest significantly higher rates of recidivism than individuals with conditional incarceration or fines. But prisions in Finland are much different than in the United States, so custodial sanctions may be as effective of a deterrent as to community sanctions when conditions of incarceration are alike to Finland (50).

Other studies argue that there is no evidence of the effect of punitive interventions as a deterrent to criminal recidivism (53–55), while there is some evidence that experiencing incarceration can be criminogenic in itself in individuals who committed substance-related crimes (54, 55). So, even if incarceration does not harm the offender’s behavior, its impact on employment, housing, family structure, and reintegration puts at risk and increases the likelihood of recidivism in PAS use and crime, due to the absence of sources of informal control and social ties (46, 53, 54).

Incarceration seems to increase the probability of recidivism, both in crimes related and unrelated to PAS (44, 51, 54). It was found that individuals sentenced to prison increase the proportion of recidivism (overall recidivism increased by 0.11 and PAS-related crimes by 0.8), with this data being statistically significant for caucasian individuals involved in the criminal justice system (51). Compared to those accused of PAS-related crimes, individuals accused of crimes related to theft seem to be 61% more likely to recidivate, and 37% more likely than individuals accused of other crimes, in 12 months (61). Individuals involved in the criminal justice system appear to be more likely to recidivate when incarcerated (53, 54). Namely, after 4 years, these individuals who commited PAS-related crimes are five to six times more likely to recidivate, and this data is statistically significant (54). Thus, individuals sentenced to incarceration are more likely to be, once again, arrested and charged with a new crime (53, 54).

Individuals sentenced to incarceration have higher rates of recidivism, and recidivate more quickly than individuals on probation (53, 54); 65% of individuals sentenced to prison for PAS-related crimes were charged with a new crime during the 4-year follow-up period vs. 36% on probation (53). Individuals sentenced to probation take longer to recidivate (53, 54), with the average number of months elapsed until a new prison offense being 18.12 vs. 33.5 on probation (53). Supervision under probation appears to reduce involvement in criminal behavior; in particular, crimes associated with methods to fund PAS use, such as shoplifting, decreased from 54%, before supervision, to 17% (48).

#### Treatment programs

Participation in a Drug Treatment Alternative to Prison (DTAP) program decreases recidivism rates, compared to individuals sentenced to prison (42, 43, 55). After 1 year, the likelihood of a new arrest is 23% in DTAP vs. 45% sentenced to prison, with this data being statistically significant (42). Program participation through DTCs appears to be equally effective in reducing recidivism (43). DTC participants who recidivate exhibit less severe offenses, in addition to the average time in jail being lower for DTC participants than for non-DTC participants (44 days vs. 126 days, *p* < 0.0001) (43). The results point to a decrease in new convictions for all types of crime among DTAP participants, with only 8% of the DTAP sample presenting a new conviction for PAS-related crimes vs. 18% of those sentenced to prison, especially for participants who finished treatment. DTAPs seem to improve public safety by involving high-risk individuals involved in the criminal justice system in treatment, including those selling PAS, who are often excluded from programs (42). Drug Courts had fewer arrest charges (*p* < 0.01) and conviction charges (*p* < 0.05) each year than participants allocated to tradicional adjudication in a 15-years analysis (62).

Compared with individuals on probation, the number of individuals on parole who finish the treatment is lower, completion of the treatment program on probation 38 vs. 24% on parole, and these are the individuals who recidivate more quickly (45). After 12 months, the arrest rate is higher for individuals on parole (38 vs. 24% on probation) (45). Regarding offenders on parole, the probability of recidivism was 1.6 times higher among those who did not receive treatment (58). New serious charges in the 12 months after the program are compared between the DTC and probation cases. DTC have less new serious charges than probationers (92.1% DTC avoid a new serious charge vs. 79.6% probationers, *p* < 0.01) (56).

The completion of programs appears to function as a protective factor in terms of criminal recidivism (56, 61). Participants who fail to complete the programs exhibit a higher risk of recidivism than those who complete it (43, 44, 52, 55, 60, 58, 61). After 3 years, 23% of DTAP participants who successfully completed the program are once again arrested vs. 52% of participants who did not complete the program (44). Participants of the MERIT, who did not complete the program, have a 50% higher risk of criminal recidivism (61). After 12 months, participants who completed the MERIT exhibit fewer charges for PAS-related crimes, theft and robbery (31% MERIT vs. 54% non-MERIT participants) (52). DTC participants who successfully complete the program are less likely to recidivate (30% DTC vs. 46% N/DTC, *p* = 0.01) (43), and avoid serious reoffense compared to participants who were unsuccessful (56). Graduates were 26.3 percentage points more successful to avoid new conviction than unsuccessful cases [χ^2^ (1, *N* = 199) = 14.39, *p* < 0.001] (56).

The risk of new arrest is 61% lower in participants who completed the RIP/Danda program, even if these programs are not statistically significant in reducing recidivism (60). In addition, completion of the programs also appears to have an impact on the severity of criminal recidivism; in particular, the 4% of participants who criminally reoffended were detained for non-violent crimes (44). Of the participants who committed more severe offenses, 69% completed the DTAP successfully vs. 74% non-participants vs. 76% who did not complete the program (44).

Individuals involved in the criminal justice system who complete the programs take longer to criminally recidivate than those who do not complete them (41, 43, 52, 55, 60). Of the individuals who completed treatment, 11.8% were convicted of a new crime vs. 29% of those who did not complete treatment. Thus, 6 months of treatment appears to be sufficient to reduce involvement in new criminal offenses (58). Participants who remain in treatment longer are less likely to reoffend than those who have been in treatment less time (43, 55). The longest time without recurrence is seen in the DTC treatment group. For those who re-committed criminal practice, the average number of days without a re-offense was 614 for program participants vs. 463 for those who did not participate in the program (43).

Over 2 years, among DTC participants, those who were referred for supervision exhibited higher recidivism rates than those who were referred for treatment (62.4% under supervision recidivated vs. 40.8% under treatment recidivated), and treatment appears to be the most consistent and most significant indicator of the longest time without criminal recidivism (41). The implementation of programs within systems, whether they are jail or prison, seems to demonstrate that, in both, the rates of treatment completion are high (65%) (40).

Thus, treatment appears to be an effective alternative in reducing criminal recidivism (40–44, 52, 55, 60, 57, 47–62).

#### Treatment programs: Practical implications

In comparison with other responses from the justice system, there is a greater number of individuals on parole who are arrested again after treatment. The reason pertains to this specific population being subjected to closer observation by law enforcement officers, which increases the likelihood of detecting individuals who are not complying with the law, compared to individuals without strict monitoring (39). As such, it is necessary to take this into account when comparing the high number of new incarcerations (39, 45, 48). Individuals involved in the criminal justice system integrated in programs, as an alternative to prison, receive more severe convictions, although the programs significantly reduce the likelihood of new charges, arrests and convictions and, thus, prove to be an effective alternative to incarceration (47). This can be understood by taking into consideration studies on probation, which report that substance-using individuals who do not comply with treatment plans, or have more severe substance-related problems, have their measure more easily revoked (48).

### Reduction of substance use

Incarceration seems to decrease PAS use, while the offender is incarcerated and even after release (49). However, the longer the time elapsed since incarceration, the greater the likelihood of using substances again (49).

Regarding TASC, it allowed the reduction of PAS use in three of the five States where it was implemented (39). The treatment of PAS use among individuals involved in the criminal justice system has shown that individuals who used cannabis have higher rates of program completion (24.3%), followed by individuals involved in cocaine use (16.2%), when they undergo residential treatment programs (40).

Individuals on probation, at a higher rate, tend to undergo outpatient treatment, 86.8 vs. 81.2%, and individuals on parole, at a higher rate, undergo residential treatment, 18 vs. 13.2%, and it is from this type of treatment that parolees tend to benefit most (45). However, individuals on probation have higher rates of program completion than individuals on parole, and treatment success, 37 vs. 24.2% on parole (45). It should also be noted that supervision under probation seems to decrease the PAS use (48). Treatment seems to be effective in reducing PAS use, namely by enabling individuals to acquire strategies that allow them to deal with it (57). However, there is a need to adopt a pragmatic approach with this type of population, while admitting that relapse is inherent to the substance (ab) use treatment process, and that professionals who work with these individuals must have knowledge about this phenomenon (48).

## Discussion

Using strict criteria and following the guidelines of PRISMA, we attempted to answer the following research question: what is the role of treatment and/or punishment, as responses of the justice system, in reducing substance use and/or criminal recidivism in individuals who use substances and commit crimes?

The results of this systematic review indicate that incarceration appears to have little impact on criminal recidivism (46, 51, 53). This finding is in accordance with what is reported in the specialized literature, and it is emphasized that punishment, *per se*, seems to be an ineffective response to the problem of PAS abuse in substance-using individuals who are also involved in the criminal justice system (36, 63, 64). The literature also points to the potential criminogenic effect^1^ that the incarceration of low-risk individuals seems to exhibit (14, 27, 36, 65, 66), in terms of employability, parenting, and the ability of these individuals to become active members of society (36), which increases the likelihood of recidivism (14, 65). Similar evidence was found in the present systematic review, which verified that the experience of incarceration can be criminogenic (54, 55). In particular, criminal recidivism can be greater with the time of incarceration (55), in addition to increasing deficits in the different areas of functioning of these substance-using individuals involved in the criminal justice system (46, 53, 54).

Individuals on probation have lower recidivism rates than individuals who are incarcerated (53, 54), which corroborates the idea that probation has a lower cost than incarceration (15) and that prison sanctions does not seem to reduce recidivism more effectively than suspended sentences (65). Specifically, the cost of supervising an individual in the community is less than the cost of keeping the individual in prison (15). In addition, supervision under probation appears to decrease involvement in criminal behavior (48, 67). Studies suggest that probation involves greater monitoring than a suspended sentence, and that existing revocations must be understood and explained, as most probationers have seen this measure overturned for moderate violations, e.g., failure to maintain employment, non-completion of the program, leaving their area of residence without permission, in addition to the fact that probation agents seem to regard failure in the program as undeserving of flexibility interventions, leading to greater revocation of cases and consequent incarceration (15, 68). Therefore, the approaches used by the supervision agencies must be taken into consideration, since the opportunity for psychological and social support this measure provides is undeniable.

Individuals placed on probation also have better program completion rates than individuals on parole (45). Nonetheless, it is important to note that probation is an alternative available in the trial phase, while parole is available in the post-sentencing phase, necessarily involving compliance with a certain period of time in incarceration (15). Furthermore, as mentioned above, a longer incarceration time is associated with greater criminal recidivism (55). Nevertheless, the results of this work also suggest that criminal recidivism among individuals placed on parole is greater in those who did not receive treatment (58).

As for treatment, the present study found that this is the most consistent and significant indicator of the longest time without recidivism (41, 58), thus making it an effective alternative in reducing criminal recidivism (40, 42–44, 55, 60, 57, 47, 61, 52). Therefore, treatment should be an alternative to incarceration, offered to substance-using individuals involved in the criminal justice system, since it is effective in reducing criminal recidivism and/or substance use (15, 17, 69). Literature on the effectiveness of criminal reactions considers that there are alternatives that appear to be more effective than others, namely: methadone and heroin treatments, therapeutic communities, psychosocial approaches, Drug Courts, supervision under probation, and parole. Thus, the benefits may be greater if certain types of interventions are prioritized over others (34).

Participation in treatment programs reduces recidivism rates compared to incarceration (42, 43, 55). As such, treatment can be a more effective alternative in promoting public safety than incarceration (42). It should also be noted that completion of programs seems to be an important indicator in terms of criminal recidivism (61, 70, 5) and its severity (44), since the failure to conclude the program increases the risk of criminal recidivism (43, 44, 52, 55, 60, 58, 61). The literature also suggests that the length of stay in treatment is also related to the likelihood of criminal recidivism (70, 5). This is corroborated by this systematic review, which found that individuals involved in the criminal justice system who spend less time in treatment are more likely to recidivate (43, 55, 70).

The present systematic review also pointed out that treatment is effective in reducing PAS use, allowing individuals to acquire strategies to deal with their substance use (57). This finding had already been highlighted in previous studies, which found that substance-using individuals involved in the criminal justice system are more likely to be re-incarcerated (36), and do not exhibit a reduction in substance (ab) use after incarceration. Among possible explanations for these findings are the causes leading to the first incarceration not having been addressed, as well as the possible existence, when individuals are released, of a set of factors that increase the risk of substance use relapse, such as social stigma and the difficulty to access legitimate employment as a means of survival (32, 36).

Research in the area of PAS use and crime is varied and largely uncoordinated (2), and the data available in the EU are often complex, fragmented, and difficult to compare (71). Despite the various guidelines and recommendations for knowledge sharing among the scientific community, most EU countries do not have data to assess the alternative interventions available, which makes it difficult to evaluate effectiveness (17). In addition, some results are based on a low number of study participants, which does not allow them to draw significant implications and conclusions (2). This situation can be explained by the absence of shared rules on data recording and collection, but also by the overlapping of structures responsible for developing the reports (71), which relates to the lack of monitoring and evaluation of the interventions applied (17). For many diversion programs, there are no methods of evaluating, collecting, and publishing data that allow the assessment of their effectiveness in terms of cost reduction and recidivism (18).

The diversity of studies, collection methods, databases, and methods of analysis are evident in this systematic review, which makes it difficult to analyze certain variables. Specifically, the analysis of substance use is difficult, so these findings should be interpreted with some reservation. Particularly, in some contexts, using a PAS represents a criminal offense, thus, results related to PAS use may appear grouped in the data about criminal recidivism. In many studies, it is not possible to disconnect one variable from the other and, therefore, to distinguish whether there is data on the reduction of PAS use, among the reduction of criminal recidivism. Furthermore, the different contexts in which these issues are applied and studied enhance the inconsistencies in the vocabulary related to some terms, namely due to the diversity of studies and laws. Thus, terms related to substance abuse, such as e.g., drug user, abuser, problem user, and addict, make the harmonization of concepts a challenge (17), and this also occurs with terms related to the type of offense, e.g., drug offenses, drug-involved offenses, drug crimes, drug-related crimes. Another constraint in the specialized literature is due to the fact that most studies are conducted in the USA (34), which is also evident in the present review, as there are more studies (*n* = 16) related to the American context and some (*n* = 29) were excluded for being theoretical studies. As such, it is important to be alert to the possibility of bias in the research (5).

The growing awareness and adoption of alternative proposals to incarceration, for substance-using individuals involved in the criminal justice system, comes from the increasing recognition of PAS abuse as an illness that requires treatment (13, 35–36, 70). Thus, PAS use should be seen as a health-related issue, and a perspective of risk reduction and evidence-based treatment should be adopted, both for the substance-using population and for individuals who have committed substance-related crimes (13). Many of the substance use/abuse disorders can be effectively treated through a variety of psychopharmacological and psychosocial interventions, in different treatment settings. Therefore, opportunities for screening and assessing disorders, namely those related to substances, must be present in all points of contact with the justice system (15, 70). It should be noted that, according to the literature, the treatment option exists at all stages of the criminal justice system (prosecution/pre-trial, trial, and post-sentence).

The results of this systematic review point to the existence of characteristics that prove to be more common among substance-using individuals involved in the criminal justice system, and that the results of the programs may be influenced by the characteristics of the individuals. Thus, it would be useful to obtain more in-depth information about the characteristics of the program participants, namely life history and motivation for change. However, studies only present basic sociodemographic data, and more detailed information about these individuals involved in the criminal justice system is lacking (34). It is, therefore, essential that a comprehensive medical and psychosocial assessment (15, 17), conducted by specialists (18), be carried out, so as to identify the services from which the offender would benefit most. This way, it would be possible to avoid incoordination and duplication of procedures within the justice system (5), by assessing the needs of the individuals and developing an appropriate treatment plan for each one (15, 17, 70), which, later, allows one to analyze which programs seem to work more with certain characteristics of the individuals. The justice system can, thus, play an important role in identifying and evaluating these individuals (15).

In addition, the services provided must be sensitive to gender issues (13, 23, 5). The present study allowed us to verify that men are more likely to recidivate (49, 54, 60), with their recidivism being more severe (46) than in women. Thus, as the literature reports, in comparison with men, women have different: patterns of use/abuse (5, 72); crime (5, 73); and social, psychological, and economic circumstances (5). For these reasons, there must be differences in the adoption of alternative interventions (28), and in the programs applied. We also suggest the use of a comprehensive and integrated approach, in order to maximize the success of the treatment and to reduce the damage within the community, without jeopardizing public safety, nor the accountability of the individuals involved in the criminal justice system (70, 5).

It is important to note that not all individuals who use substances need treatment; in case they do need treatment, evidence-based treatment should be offered as an alternative to incarceration, and when there is no addiction, they should be referred to harm reduction services (13, 70). However, self-referral remains the most common route for specialized treatment for the use/abuse of PAS. In Europe, the treatment of PAS addiction is predominantly performed on an outpatient basis, with specialized treatment centers being the largest providers of care for individuals who use PAS. As an example, in 2017: (i) 973,000 individuals who used PAS received outpatient treatment, namely in specialized treatment centers; (ii) 64,000 individuals who used PAS underwent an inpatient regime, namely residential hospitalization; (iii) and 81,000 individuals received treatment in prison (74).

In short, alternative interventions to punitive policies, namely treatment, seem to provide useful tools in solving problems of different orders for these individuals, including at the personal and social levels. However, for the treatment to be adequate and meet the expected goals, it is necessary that the strategy and intervention plan be specific to that user. Thus, it is pertinent to take into account the characteristics of each individual and, specifically, the link between the needs of the individuals involved in the criminal justice system, their problems, and resources (15, 17, 70, 5), and the intervention programs available (17). Given the specificity of this population, there does not seem to be a single model for intervention, since no treatment is exceptional and universally applicable. Thus, the type of treatment, its modalities, as well as restrictions and levels of supervision, must be assessed on a case-by-case basis (70, 5). As such, treatments must be available and accessible, as well as attractive and appropriate for the needs of those who attend them. In addition, ethical standards in the treatments provided must be ensured and evidence-based (15). Therefore, it is important to invest in the development and evaluation of all types of treatment, especially those that demonstrate effectiveness (74).

Alternative interventions to incarceration vary from context to context and over time, so it is difficult to identify them in practice (17). It is important to take into account the context where the alternatives are implemented, since an alternative can work well in one context and fail in others. Thus, the capacity of the institutions and the way they function, as well as cultural differences, public opinion about alternatives to incarceration, and restrictions in terms of legislation and budgets must be taken into account (18). Likewise, there are alternatives that may work for some individuals involved in the criminal justice system and not for others (1). The key to the success of alternative interventions to punishment/incarceration seems to be the availability of a variety of interventions that can be adapted to the needs of substance-using individuals involved in the criminal justice system, with different types and levels of substance-related problems (26).

The justice and treatment systems are effective when they work together for the successful treatment of substance-using individuals involved in the criminal justice system (13, 44, 48). Therefore, cooperation efforts between the various justice departments and the joint elaboration of programs are needed (13, 39, 58, 48), in order to guarantee that low-risk individuals involved in the criminal justice system do not overburden or cripple the criminal justice systems. Cooperation between services allows the provision of health care, risk reduction and treatment, and/or ensures social and/or economic conditions for this specific population, in order to provide support for the prevention of recidivism and the promotion of social reintegration (13). Treatment should be promoted through coordination between the criminal justice system and social services (15).

Across Europe, crimes related to substances need intervention, and this type of crime is responsible for the incarceration of 10–25% of inmates with a final and unappealable sentence. The psychological and social impact of incarceration is undeniable, although it also becomes an opportunity for the development of programs aimed at removing problematic substance use. The transversality of the problem, in national and European terms, demonstrates the need to promote substance treatment in prisons, both due to the association of this problem with public health issues or because of the trajectories of poverty and social exclusion to which these substance-using individuals involved in the criminal justice system tend to be subjected (24). It is crucial to ensure the continuity of the treatments provided, entry and release of incarcerated individuals, so that the treatment benefits initiated during incarceration be maintained when individuals are released. Thus, contacts should be made with health and social services for the reintegration of the offender into society, valuing work within a network (23).

In short, treatment is effective in reducing criminal recidivism and/or substance use, with the risk of arrest being lower in individuals involved in the criminal justice system who complete the programs, even if these do not show statistically significant results in terms of criminal recidivism (60). Therefore, priority should be given to adopting alternative interventions to incarceration, namely interventions that include treatment, with the need for continuous scientific evaluation, monitoring, and publication that allow data collection. It is also essential to adapt treatment to each case.

## Conclusion

The present systematic review allowed us to conclude that punishment shows little impact in reducing criminal recidivism and/or PAS use. Treatment, in turn, presents itself as an effective response in reducing substance use and/or criminal recidivism.

Despite the recommendations of several organizations, in order to find alternatives to incarceration/punishment for substance-using individuals, there are still many barriers to treatment. We refer to barriers related not only to the lack of recognition of the relevance of treatment, but also to the scarcity of economic resources, infrastructure, and means necessary to serve this specific population, as well as technical personnel with knowledge about substance-related issues (36).

In conducting this systematic review some difficulties were faced, which are mainly related to: (i) the fact that studies on alternative interventions to punishment are conducted mainly in the USA, which implies approaches to the problem that are different from those adopted in the EU; (ii) the existence of different terms and concepts, as well as laws and specificities related to the subject under study; and (iii) the lack of rigorous evaluation, monitoring, and publication of alternative interventions to punishment, namely in the EU.

In addition, this review has some limitations, which are related, in particular, to a possible bias in the information collected: (i) potentially biased factors listed in the articles analyzed: number of sample (41, 57); variables (41, 47, 54); group comparison (43, 48, 53); samples (48); logistical and methodological concerns (44, 46, 48, 58, 43); characteristics of the participants (45, 48, 61, 58); contextual and demographic factors (45, 48, 57), type of consumed substances (61); judge’s decision regarding the type of sentence (53); omitted data (45); (ii) the systematic review itself can contribute to bias related to selection, selective outcome reporting, clinical or statistical inconsistency, and imprecision that may lead to systematic and random errors (75, 76). The non-performance of the quality table is also a limitation of the present study, this quality assessment would explain the evaluation of the methodological quality of studies and is recommended to integrate systematic reviews. However, this evaluation was not elaborated and so it may influence the robustness of the paper.

Despite the obstacles and limitations presented, the present systematic review allowed us to analyze what has already been published in the literature on the subject under study, and the compilation of this information made it possible, in turn, to draw some conclusions, of which the following stand out: (i) incarceration is not an effective alternative for substance-using individuals involved in the criminal justice system, and may even have a criminogenic effect on them; (ii) treatment seems to be the most effective alternative for reducing substance use and criminal recidivism; and (iii) treatment success is associated with participation, length of stay, and conclusion of programs. PAS use continues to be a socially stigmatized behavior that, at times, is not regarded by the justice system under a medical approach, which does not allow the guarantee of treatment, as happens for other medical conditions (36). The growing awareness that substance abuse/addiction must be understood as a health issue can foster greater openness and referral for treatment. In this referral process, the evaluation of these individuals involved in the criminal justice system is of special importance, recognizing that there is no single model/program that is effective for all substance-using individuals involved in the criminal justice system, given the heterogeneity of these populations. It is, therefore, essential to take into account the individual characteristics, type and levels of problems related to PAS abuse/addiction, for the success of the treatment.

Nevertheless, there is still a long way to go with regard to alternative interventions to incarceration. Despite studies, over time, pointing to the ineffectiveness of incarceration, responsible crimes directly and indirectly related to PAS are for 15–20% of incarceration in Portugal (77). In reality, there remains a need to monitor and evaluate the effectiveness of alternative approaches to punishment, as well as better documentation and recording of practices associated with them, so as not to miss opportunities for change and future improvements (17). Specifically, given the potential of treatment, it is urgent to evaluate the programs that are being implemented, as well as the characteristics of their participants. It is also important to understand with which participants the different programs seem to be more successful. Similarly, it is necessary to recognize that treatment policies, services, procedures, and approaches must be constantly monitored and evaluated (15, 33). In fact, it is important to emphasize the need to adapt the programs to the needs and individual characteristics of each offender in order to achieve a successful intervention.

There is a greater need than ever to adopt PAS policies based on reliable scientific data (71). This is also materialized by the growing incentive and challenge, on the part of formal institutions with connections to the States, to seek and adopt alternative interventions to incarceration/punishment in the case of substance-using individuals involved in the criminal justice system. Thus, the present study helps establish starting points for the evolution of the interventions adopted until then, working toward enhancing treatment, instead of incarceration, in substance-using individuals involved in the criminal justice system. This seems all the more important if we take into account that not treating individuals involved in the criminal justice system with substance-related problems is a missed opportunity to promote public health and safety (32). Thus, integrating treatment into the criminal justice system allows it to reach individuals who, otherwise, would not have access to it (36).

## Data availability statement

The raw data supporting the conclusions of this article will be made available by the authors, without undue reservation.

## Author contributions

All authors listed have made a substantial, direct, and intellectual contribution to the work, and approved it for publication.

## Footnotes

1 Criminogenic effect is used to represent the effect that a sanction can be, in itself doubly punitive, generating effects contrary to the intended. This means that there is a risk of aggravating criminal behavior when we apply a punitive measure, either by insertion in the prison subculture, desocialization due to the breakdown of professional, family, and social relationships or by issues related to the prejudices existing in society about the incarcerated population. The combination of these factors can culminate in an effect opposite to what is desired and thus potentiate criminal behavior rather than cease it.
